# An Over 90 dB Intra-Scene Single-Exposure Dynamic Range CMOS Image Sensor Using a 3.0 μm Triple-Gain Pixel Fabricated in a Standard BSI Process †

**DOI:** 10.3390/s18010203

**Published:** 2018-01-12

**Authors:** Isao Takayanagi, Norio Yoshimura, Kazuya Mori, Shinichiro Matsuo, Shunsuke Tanaka, Hirofumi Abe, Naoto Yasuda, Kenichiro Ishikawa, Shunsuke Okura, Shinji Ohsawa, Toshinori Otaka

**Affiliations:** Brillnics Japan Inc., 6-21-12 Minami-Oi, Shinagawa-ku, Tokyo 140-0013, Japan; yoshimura.norio@brillnics.com (N.Y.); mori.kazuya@brillnics.com (K.M.); matsuo.shinichiro@brillnics.com (S.M.); tanaka.shunsuke@brillnics.com (S.T.); abe.hirofumi@brillnics.com (H.A.); yasuda.naoto@brillnics.com (N.Y.); ishikawa.kenichiro@brillnics.com (K.I.); okura.shunsuke@brillnics.com (S.O.); ohsawa.shinji@brillnics.com (S.O.); otaka.toshinori@brillnics.com (T.O.)

**Keywords:** CMOS image sensor, dynamic range, single exposure, CMOS image sensor pixel

## Abstract

To respond to the high demand for high dynamic range imaging suitable for moving objects with few artifacts, we have developed a single-exposure dynamic range image sensor by introducing a triple-gain pixel and a low noise dual-gain readout circuit. The developed 3 μm pixel is capable of having three conversion gains. Introducing a new split-pinned photodiode structure, linear full well reaches 40 ke^−^. Readout noise under the highest pixel gain condition is 1 e^−^ with a low noise readout circuit. Merging two signals, one with high pixel gain and high analog gain, and the other with low pixel gain and low analog gain, a single exposure dynamic rage (SEHDR) signal is obtained. Using this technology, a 1/2.7”, 2M-pixel CMOS image sensor has been developed and characterized. The image sensor also employs an on-chip linearization function, yielding a 16-bit linear signal at 60 fps, and an intra-scene dynamic range of higher than 90 dB was successfully demonstrated. This SEHDR approach inherently mitigates the artifacts from moving objects or time-varying light sources that can appear in the multiple exposure high dynamic range (MEHDR) approach.

## 1. Introduction

High image quality in low light conditions is highly appreciated in many applications, such as security, surveillance, automotive, and IPC applications, to name a few. On the other hand, high sensitivity to low-light objects is also required in these applications. In low light conditions, image sensors are usually operated in a high gain mode, and image quality suffers from low signal saturation due to the limited dynamic range. Many approaches have been introduced in order to expand the image sensor dynamic range while keeping low light image quality, including multiple exposure [1,2,3,4], logarithmic compression [5,6], knee compression [7], multiple column gain readout [8], and more [9,10]. 

Among these approaches, the multiple exposure high dynamic range (MEHDR) approach is the most common scheme, where images with different exposure times are captured and then merged into a high dynamic range image. Line interleave exposure control or pixel interleave exposure control schemes are also included in this category. However, this approach has fundamental issues associated with the different exposure timings for long exposures and for short exposures, which can generate artifacts against moving objects or time-varying light sources like pulsed LEDs. Another issue is SNR degradation at the conjunction point of the two images. To suppress the artifacts from the pulsed LEDs, chopping integration has been introduced [11]. However, even with the chopping integration, the SNR drop at the conjunction point is still an issue.

The second major approach uses two kinds of pixel—one with lower sensitivity, and the other with higher sensitivity—implemented in the same pixel array, and the different sensitivity signals combined into a linear signal [12]. This approach needs redundant pixels. Also, it needs careful optical design to obtain uniform optical performance of angular dependence, cross-talk and spectral response between the two kinds of pixels.

An ideal solution is to expand pixel saturation while keeping high sensitivity in low light conditions. The lateral overflow integration capacitor (LOFIC) approach is considered to be one of these solutions, with a high dynamic range of more than 100 dB having been demonstrated in a single exposure [13,14]. On the other hand, LOFIC needs a charge accumulation node outside the photodiode. Reduction of junction leakage and defect control of the charge accumulation node are challenging issues in terms of manufacturability.

The basis of the dynamic range enhancement introduced in this image sensor is a dual gain readout scheme [15], and follows the following sequence: (1) a photodiode signal is read out in a high gain mode, and (2) read out in a low gain mode; then, (3) the two signals are merged into a high dynamic range linear signal. The intra-scene dynamic range in this scheme is defined as the ratio between linear full well (LFW) to readout noise in the high gain condition. Therefore, increase of the LFW and reduction of readout noise floor are required to increase dynamic range.

In this paper, we present a 3 μm, high dynamic range pixel, using a multiple-junction photodiode and a triple-conversion gain configuration [16] in Section 2. The high gain conversion of the pixel is able to detect small signals in low light conditions with a low readout noise, while low gain conversion covers a large LFW of 40 ke^−^. In Section 3, the architecture of the image sensor and pixel operation sequence are described, followed by the characterization results [17] in Section 4, and conclusions in Section 5.

## 2. The Triple-Conversion Gain Pixel

In this section, pixel configuration, photodiode design and operation sequence of the triple-conversion gain pixel are described.

### 2.1. Pixel Configuration

Electron-referred readout noise can be reduced by increasing pixel conversion gain. However, higher pixel conversion gain results in lower signal saturation, because of the limited voltage window of the charge detection node.

**Figure 1:**
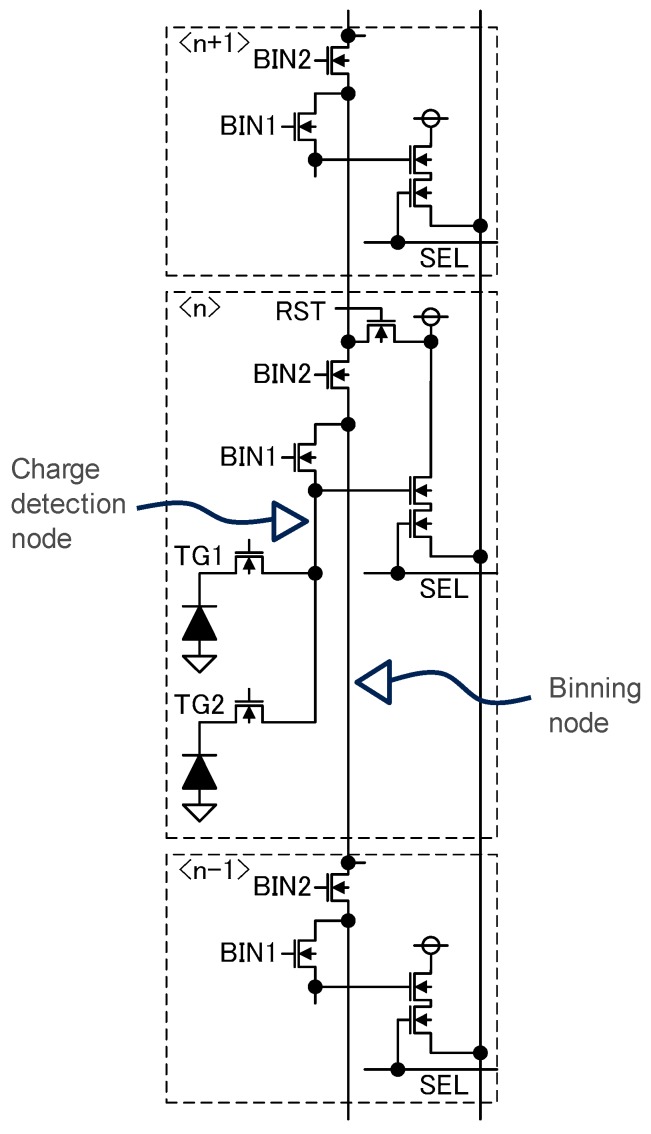
Configuration of the triple gain pixel. RST transistors in top/bottom neighboring pixels are not drawn.

The developed 3.0 μm pixel configuration is shown in Figure 1. The pixel has a two row-shared structure. Through switches, BIN1 and BIN2, the charge detection node is vertically connected. These binning switches act as vertical binning switches. To prevent extra parasitic capacitance when high pixel gain is required, the charge detection node is isolated from the vertical binning node by the BIN1 switch.

The BIN1 and the BIN2 switches are utilized not only for the binning purpose, but also for pixel gain control. When the BIN1 switch is closed during the pixel readout, the gate capacitance of the BIN1 switch and metal capacitance of the vertical binning line serve as an additional capacitance of the charge detection node and thus reduce the pixel gain. Closing the BIN2 switch additionally, more capacitance is added to the charge detection node. Thus, by controlling the BIN1 and the BIN2 switches, three different conversion gains can be obtained without additional devices like a poly-insulator-poly (PIP) capacitor or a metal-insulator-metal (MIM) capacitor, which is used in the conventional dual-conversion gain pixel architecture [18].

### 2.2. Dual Gain Readout Sequence

**Figure 2:**
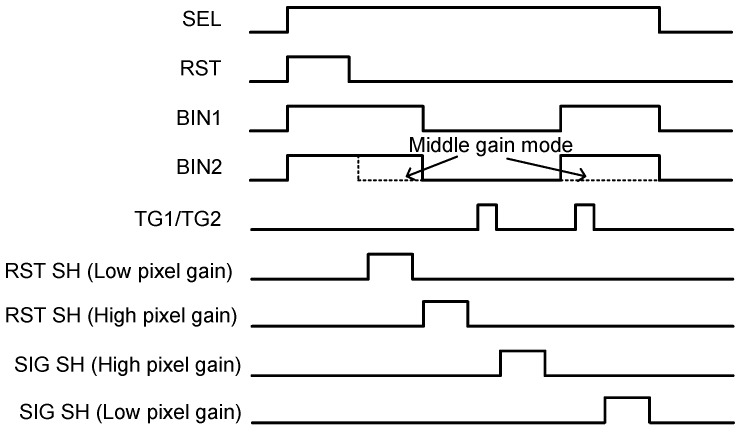
Pixel and switch control timing sequence for dual gain pixel readout with a combination of LPG mode and the HPG mode. When BIN2 switch timing is changed to the dotted line, the middle pixel gain MPG signal is obtained.

To obtain a linearized high dynamic range signal, the same photodiode signal is read out twice with different pixel gains. Figure 2 shows a pixel operation timing for the dual pixel gain readout. Firstly, RST, BIN1 and BIN2 switches are all turned on to initialize voltage of the vertical binning node and the charge detection node. After the RST switch is turned off, a low pixel gain (LPG) offset signal is sampled as RST SH (Low pixel gain). Next, BIN1 and BIN2 switches are turned off, and high pixel gain (HPG) offset, RST (High pixel gain) is sampled. A first charge transfer pulse is applied on the transfer gate, TG; then, the HPG signal, SIG SH (High pixel gain) is sampled. LPG signal is obtained after BIN1 and BIN2 are turned on again, and sampled as SIG SH (Low pixel gain). Before the LPG signal readout, the second transfer pulse is applied to the TG gate so that remaining charge in the photodiode is transferred to the charge detection node in case of high light conditions. When BIN1 and BIN2 switches are turned on again, offset charge in the charge detection node is re-combined to the same condition as the RST SH (Low pixel gain); therefore, correlated double sampling (CDS) is realized for LPG signal readout as well as the HPG. When the BIN2 switch is turned off (dotted line in Figure 2) during the signal readout period, the pixel operates in the middle pixel gain (MPG) condition.

### 2.3. High Linear Full Well (LFW) Photodiode

**Figure 3:**
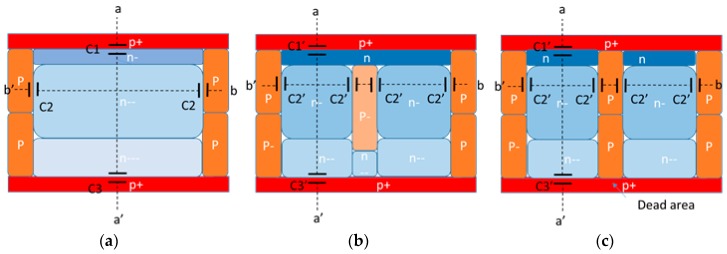
Schematic cross-sectional view of (**a**) conventional PPD; (**b**) proposed PPD; and (**c**) conventional split PPD.

A conventional pinned photodiode (PPD [19]) structure, the proposed photodiode structure and a conventional split-pinned diode structure are shown in Figure 3a–c, respectively. In the presented structure, Figure 3b, a P-type layer is formed in the center of PPD, like a P-type pixel isolation layer. Because of the narrow photodiode effect, the split photodiode structure is effective for maintaining a low *V_pin_* when the LFWC is increased by additional dosage for the N-type photodiode. The conventional split photodiode structure [20], which has 2 sets of PPDs and a transfer gate and one floating diffusion node per pixel, is shown in Figure 3c for comparison. The stratified PPD structure [21], which has vertically stacked P-type layers, is considered a reference of this type of PPD structure. 

**Figure 4:**
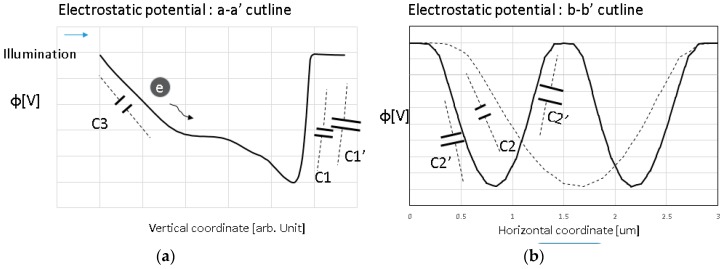
Schematic potential diagram (**a**) a-a’ and (**b**) b-b’ in the conventional PPD structure of Figure 3a and the proposed structure of Figure 3b.

Figure 4 shows an example of electrostatic potential profiles with capacitance components along the lines of Figure 4a a-a’ and Figure 4b b-b’ in Figure 3a for both the conventional PPD and the presented one. In the simple P^+^/N step junction model, the depletion layer width *W_d_* in PPD is expressed as Equation (1), where ε is the semiconductor dielectric constant, *V_pin_* the pinning voltage when the PPD is fully depleted, *N_d_* the *N* dopant concertation, and *φ_bi_* the built-in potential.

(1) $$W_{d}=\sqrt{\frac{2\epsilon \left(\varphi_{bi}+V_{pin}\right)}{qN_{d}}}$$

Equation (1) suggests that the *W_d_* (fully depleted region) becomes narrower in the presented structure in Figure 3b due to the additional P-type layer between the split PPDs. Thus, the *V_pin_* to fully deplete the PPD can be lower than for the conventional structure shown in Figure 3a, even with the same *N* dopant, *N_d_*. This means that the proposed structure allows *N_d_* to be increased in order to boost LFWC with the same *V_pin_* as that of the conventional PPD.

**Figure 5:**
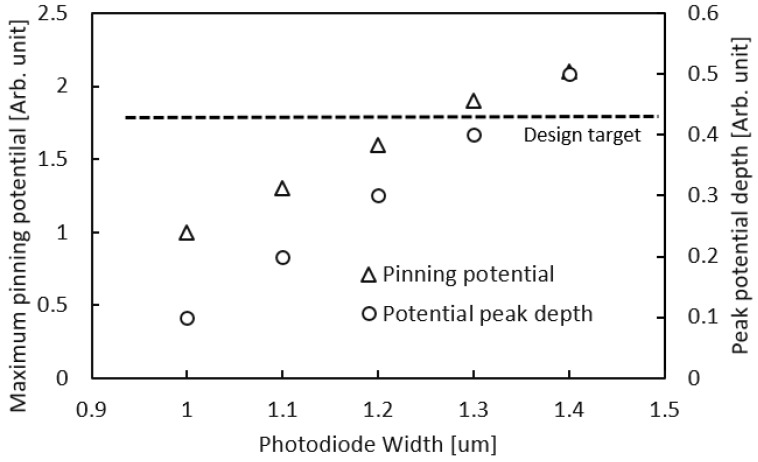
2-D device simulation result of maximum pinning potential and its depth.

Figure 5 shows the PPD width dependence of the pinned potential and the peak potential depth, as obtained by a 2D device simulation. The results show that the location of the potential peak shifts deeper and the peak potential increases as the PPD width is increased. This suggests that it is difficult to boost LFWC with a large PPD by increasing the *N* dopant without changing *V_pin_* or supplying voltage. This is a benefit of the presented PPD structure, which only uses a narrow PPD width.

**Figure 6:**
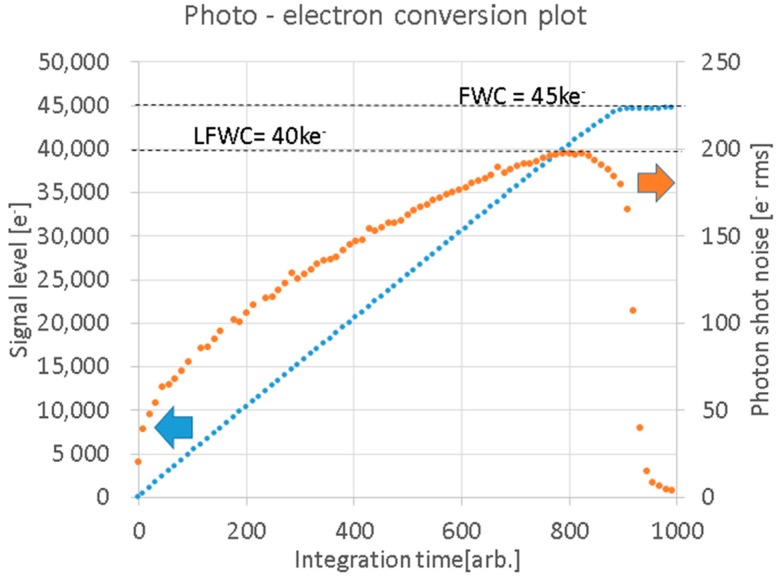
Photo-electron conversion and random noise characteristics of the pixel measured in test mode. Exposure control was done by integration time under a DC light source. Random noise basically follows the optical shot noise model. Linear full well capacity (LFWC) is defined at the peak shot noise point.

A photo-electron conversion plot for this pixel is shown in Figure 6. A linear full well capacity (LFWC) of 40 ke^−^ and a full well capacity (FWC) of 45 ke^−^ were obtained with good linearity. In this paper, LFWC is defined as signal electrons measured at the shot noise peak point. Above LFWC, random noise decreases because of pixel saturation. Also, FWC is defined as signal electrons corresponding to PD saturation. 

## 3. Sensor Architecture

**Figure 7:**
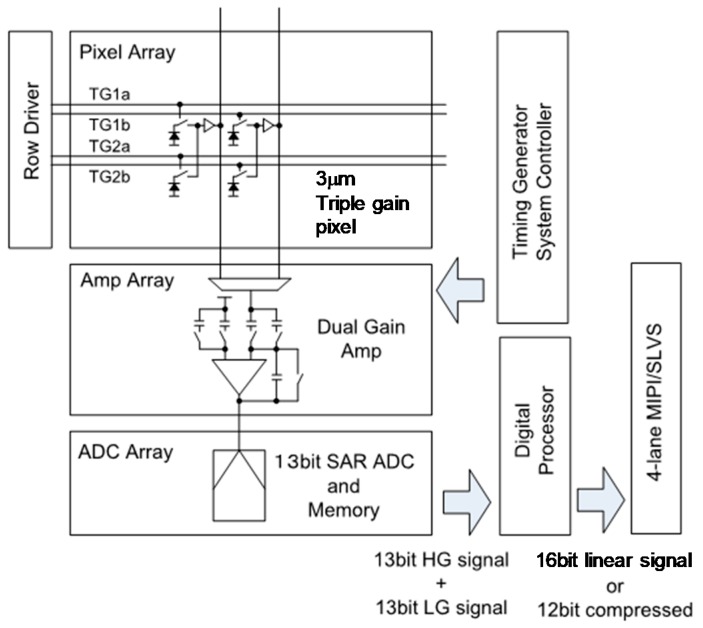
Sensor architecture. To read out even column pixels and odd column pixels serially, two TG bus lines (TG1a/TG1b and TG2a/TG2b) are introduced in a row.

Top architecture of the image sensor is shown in Figure 7. Odd column pixels and even column pixels have individual TG control lines TG1a/TG1b and TG2a/TG2b, so that even and odd column pixels are read out serially, which allows us to assign one signal chain for two pixel columns.

The column signal chain consists of an amplifier array, a 13 bit successive approximation register (SAR) ADC array and column memory. The column amplifier has two separated signal input paths, which enables two independent CDS operations with different gains. In combination with the multiple-gain pixel and the dual-gain column amplifier, flexible control with a wide gain ratio is available. For the LPG readout, an amplifier gain of either 1× or 2× can be chosen, and for the HPG readout, amplifier gains of 1×, 2×, 4×, 8× are selectable.

After A/D conversion, the two sets of signals are fed to an on-chip digital processor, yielding a high dynamic range 16 bit linear signal. Finally, the 16 bit linear signals or compressed 12 bit signals are output through 4-lane MIPI or SLVS interface. The image sensor is capable of operating at 120 fps in a normal signal readout mode, or 60 fps in the SEHDR mode.

## 4. Fabrication and Characterization Results

**Figure 8:**
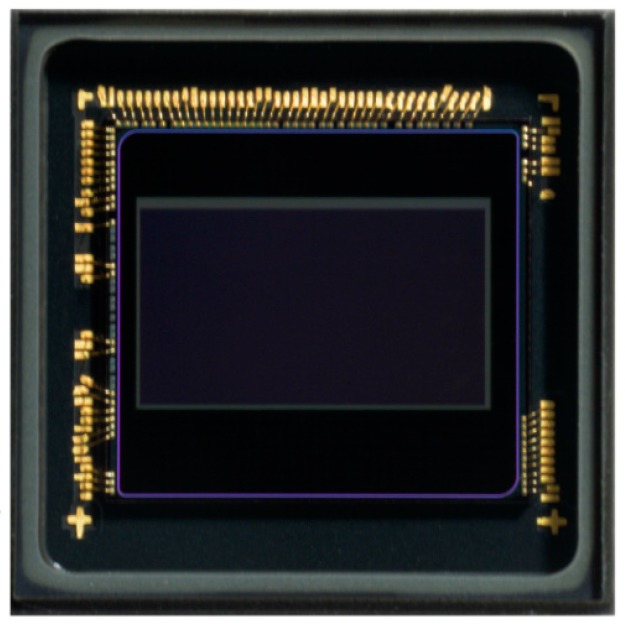
Chip photograph. Assembled in a 48 pin PLCC package.

The sensor is fabricated in a 65 nm BSI CMOS image sensor process, and assembled in a 48-pin PLCC package as shown in Figure 8.

**Figure 9:**
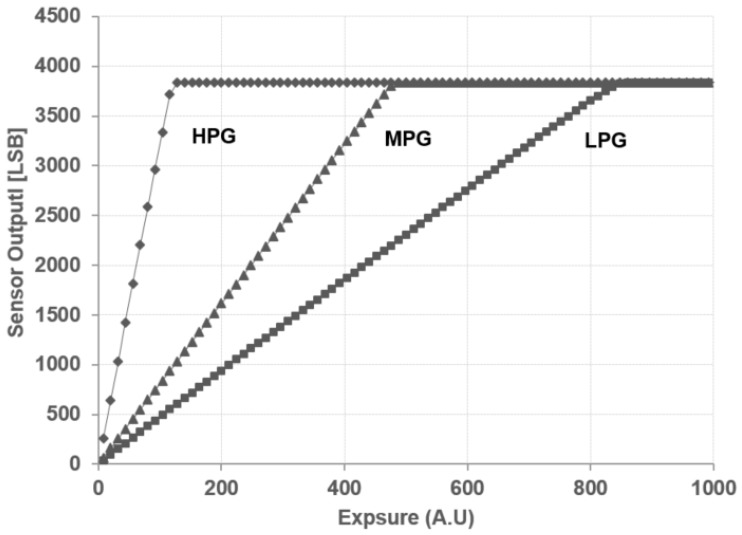
Photo conversion characteristics measured in the HPG, MPG and LPG modes. Analog gain is fixed at 1×.

Photo conversion characteristics in the normal mode are shown in Figure 9. Pixel conversion gains in the HPG condition, the MPG condition and the LPG condition were measured as 152 μV/e^−^, 38 μV/e^−^ and 21 μV/e^−^, respectively. Using the MPG as reference for a base pixel gain, the pixel gain ratio is 4.0:1:0.57.

**Figure 10:**
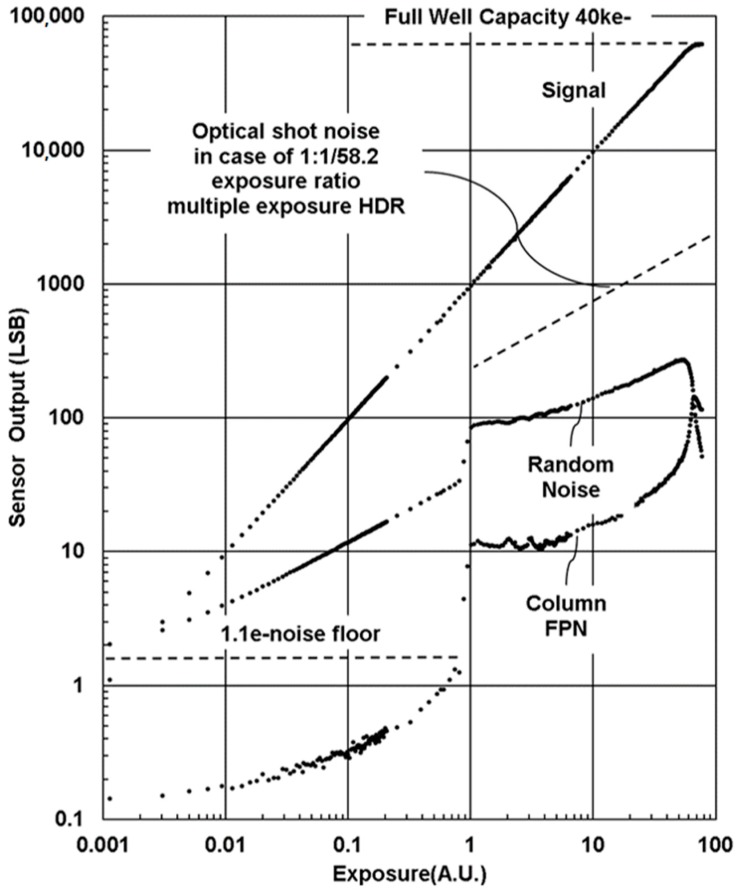
Photo conversion characteristics in the SEHDR mode. The 16-bit linearized output signal, random noise and column FPN are plotted. Gain combination is LPG + Analog gain 1× and HPG + Analog gain 8×, thus gain ratio of 58.2×.

Figure 10 shows an example of photo conversion characteristics obtained under the SEHDR mode with a gain setting of [LPG + Analog gain of 1×]/[HPG + Analog gain of 8×]. Effective gain ratio between the two signals is about 1:58.2. Dark noise floor in the SEHDR mode is identical to the noise at the highest gain setting of 1.1 e^−^. The ADC window was set at 40 ke^−^, so that maximum dynamic range of 91 dB was obtained. The number of signal electrons at the conjunction point of the HPG signal and the LPG signal was about 800 e^−^.

The signal exhibits linear characteristics in the whole range from lower than 1e^−^ to the saturation level, suggesting that the charge transfer error from the photodiode to the charge detection node is smaller than 1 e^−^, and therefore that the sensor shows excellent low light linearity.

Random noise, pixel FPN and column FPN are also plotted in Figure 10. Column FPN in low exposure conditions is lower than 1/20 of the random noise, and one can hardly see it. When the amplifier gain is changed at the conjunction point, excess noise and excess FPN are generated due to its circuit operation, and the excess random noise affects SNR by −8 dB. Except for the excess noise, random noise characteristics follow the shot noise model of ∝*S*^−0.5^ throughout the whole range, where S is averaged signal. In comparison to the MEHDR scheme, no significant noise at the conjunction point is seen. When we suppose 1:1/32 integration time ratio in the MEHDR scheme, shot noise degrades about 15 dB at the conjunction point as illustrated by the dotted line in the figure. This is one of benefits of the SEHDR scheme, because no signal charges are lost.

The photo response non-uniformity (PRNU) of the sensor is lower than 0.5%, which suggests that the process variations of the gate capacitance and parasitic metal capacitance are small enough to be used for pixel capacitance.

**Figure 11:**
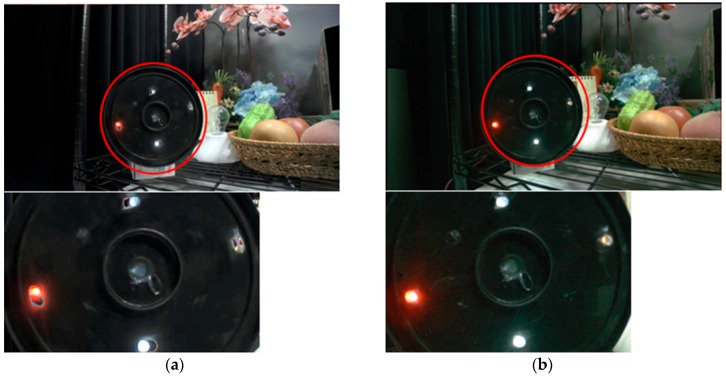
Comparison of captured images of a rotating LED plate; (**a**) two-exposure MEHDR images and (**b**) SEHDR images.

Figure 11a,b shows images captured in the conventional MEHDR mode and in the SEHDR mode reported in this paper, respectively. Motion artifacts and LED flicker artifacts are clearly suppressed in the single-exposure image.

## 5. Conclusions

An image sensor that has an SEHDR feature was reported. By introducing a triple-conversion gain pixel and a dual-gain readout scheme, the sensor is able to cover an intra-scene dynamic range wider than 87 dB in MPG + HCG mode, and 91 dB in LPG + HCG mode. Because of there being no signal loss in high light conditions, there is no significant SNR drop at the conjunction point. In addition, the SEHDR scheme mitigates moving artifacts or flicker artifact in high dynamic range image applications. Specs and performance are summarized in Table 1.

## Figures and Tables

**Table 1:** Specifications and performance.

Optical Format	1/2.7”
Pixel size	3.0 µm
Effective pixels	1928 × 1088 pixels
Frame rate	120 fps (Normal mode)
	60 fps (SEHDR mode)
Digital output	12-bit (normal mode)
	16-bit or compressed 12-bit
	(SEHDR mode)
Interface	MIPI 4 lanes, SLVS 4 lanes
Max readout signal	40 ke^−^ (LPG condition)
Responsivity	24 ke^−^/lx∙s (5100 k light source, with IR-Cut)
Peak QE in Green	78.8% (w/o glass)
Lag	less than detectable limit
Dynamic Range	91 dB (SEHDR mode, LPG/HPG)
	87 dB (SEHDR mode, MPG/HPG)
	>120 dB (MEHDR)
Readout noise	<1.1 e^−^_rms_ (max gain)
Column FPN	0.045 e^−^_rms_ (max gain)
Power	280 mW (120 fps)
